# Health service underutilization and its associated factors for chronic diseases patients in poverty-stricken areas in China: a multilevel analysis

**DOI:** 10.1186/s12913-021-06725-5

**Published:** 2021-07-18

**Authors:** Haiyan Hu, Weiyan Jian, Hongqiao Fu, Hao Zhang, Jay Pan, Winnie Yip

**Affiliations:** 1 HEOA Group, West China School of Public Health and West China Fourth Hospital, Sichuan University, No. 16, Section 3, Ren Min Nan Road, Chengdu, 610041 China; 2 Institute for Healthy Cities and West China Research Center for Rural Health Development, Sichuan University, No. 16, Section 3, Ren Min Nan Road, Chengdu, 610041 China; 3 School of Public Health, Peking University, No. 38, Xueyuan Road, Haidian District, Beijing, 100871 China; 4 Harvard T.H. Chan School of Public Health, No. 665 Huntington Avenue, Boston, MA 02115 USA

**Keywords:** Health service utilization, Underutilization, Chronic diseases, Hypertension, Diabetes mellitus, Rural, Poverty, China

## Abstract

**Background:**

Underutilization of health services among chronic non-communicable disease sufferers, especially for hypertension (HBP) and diabetes mellitus (DM), was considered as a significant contributing factor to substantial cases in terms of both avoidable morbidity and mortality. However, evidence on health services underutilization and its associated factors in poverty-stricken areas remain scarce based on previous literature. This study aims to describe health services underutilization for people diagnosed with chronic diseases in impoverished regions and to identify its associated factors, which are expected to provide practical implications for the implementations of interventions tailored to the specific needs of disadvantaged residents in rural China to achieve effective utilization of health services in a timely manner.

**Methods:**

Data were collected from a cross-sectional survey conducted through face-to-face interviews among 2413 patients from six counties in rural central China in 2019. The Anderson behavioral model was adopted to explore the associated factors. A two-level logistic model was employed to investigate the association strengths reflected by adjusted odds ratios (AOR) and 95% confidence intervals in forest plots.

**Results:**

On average, 17.58% of the respondents with HBP and 14.87% with DM had experienced health services underutilization during 1 month before the survey. Multilevel logistic regression indicated that predisposing factors (age), enabling factors (income and a regular source of care), and need factors (self-reported health score) were the common predictors of health service underutilization both for hypertensive and diabetic patients in impoverished areas, among which obtaining a regular source of care was found to be relatively determinant as a protective factor for health services underutilization after controlling for other covariates.

**Conclusions:**

Our results suggested that the implementation of a series of comprehensive strategies should be addressed throughout policy-making procedures to improve the provision of regular source of care as a significant determinant for reducing health services underutilization, thus ultimately achieving equal utilization of health services in impoverished regions, especially among chronic disease patients. Our findings are expected to provide practical implications for other developing countries confronted with similar challenges resulting from underdeveloped healthcare systems and aging population structures.

**Supplementary Information:**

The online version contains supplementary material available at 10.1186/s12913-021-06725-5.

## Background

Underutilization of health services can be defined as the failure to adopt an affordable health service that is highly possible to improve the quality or quantity of life [1, 2]. It is manifested behaviorally as not seeking medical care when feeling ill or suspecting they should go [3–5], which has been considered as a significant contributing factor to substantial cases in terms of both avoidable morbidity and mortality [1, 2]. Based on the current literature, most of the studies focused on investigating various factors associated with health services underutilization were targeted at specific types of population groups, such as pregnant women [6], dental patients [7], men receiving fertility evaluation [8], randomly selected household members [5], and hospice patients [9]. However, current studies in this field merely provided limited evidence on objective factors and residents’ self-reported factors associated with health services underutilization, especially among chronic non-communicable disease survivors.

Chronic non-communicable diseases are significant causes of mortality and morbidity, which have posed considerable threats to public health. Estimates from the Global Burden of Diseases (GBD) Risk Factors Collaborators 2019 suggested that in 2019, high systolic blood pressure, accounting for 10.8 million (95% uncertainty interval [UI] 9.51–12.1 million) deaths (19.2% [95% UI 16.9–21.3%] of all deaths), ranked as the leading Level 2 risk factor for attributable deaths in a worldwide range [10]. Furthermore, high systolic blood pressure and high fasting plasma glucose, with annualized change rates exceeding 0.5%, remain two leading causes of attributable DALYs (disability-adjusted life-years) [11].

These chronic non-communicable diseases, especially hypertension (HBP) and diabetic mellitus (DM), require routine monitoring to prevent the disorders from progressing to life-threatening exacerbations and complications [12, 13]. Appropriate and continuous management of chronic non-communicable diseases has been highlighted as the key to minimizing potentially avoidable hospital admissions due to worsening the condition among patients with chronic illnesses. Therefore, strategies should be addressed in an attempt to facilitate residents’ utilization of health services in a timely manner, thus ultimately achieving improved healthcare outcomes and reduced medical costs [14, 15]. Previous studies have identified various factors as significant determinants for health services underutilization among chronic non-communicable sufferers. For example, Bovet, et al. examined factors associated with poor health service utilization among 540 hypertensive patients in Dar es Salaam. They reported that older age was a protective factor of health services underutilization [16]. In another study, Newman et al. [17] examined the underuse of medications among US outpatients diagnosed with hypertension and diabetes, which indicated that female diabetic patients presented a higher tendency for undertreatment with guideline-directed therapeutic schemes than males.

China has launched a nationwide healthcare system reform in 2009, which has highlighted the implementation of a series of interdependent strategies as a priority, including increasing the provision of public health services, strengthening primary healthcare, providing reimbursement for beneficiaries under the coverage of different health insurance programs as well as reallocating both financial and healthcare-related human resources into underprivileged areas and for vulnerable populations (e.g., senior adults with low income) [18]. Since the initiation of this reform, substantial improvements have been achieved in improving national residents’ accessibilities to health services, thus leading to improved health services utilization in a nationwide range [19].

Despite the improved accessibility to healthcare services in the past decade, it was estimated that the likelihood of not seeking medical care when feeling ill among Chinese residents reached 25.13% in 2016 [19]. It has been recognized that failure to seek medical treatments when necessary in a timely manner is a considerable obstacle for achieving effective chronic disease management, which might lead to even more severe complications [15]. It is noteworthy that economically disadvantaged population groups tend to be more vulnerable under the impact of illness-related financial burdens, resulting in medical-cost-induced poverty due to unaffordable out-of-pocket expenses [20].

Social health insurance schemes have been established to improve financial protection for its population [21]. China’s social health insurance schemes include the Urban Employee Basic Medical Insurance (UEBMI; launched in 1998), the New Rural Cooperative Medical Scheme (NRCMS; launched in 2003) and the Urban Resident Basic Medical Insurance (URBMI; launched in 2007). Considering that urban and rural residents were enrolled in separate programs and the former population group enjoyed greater benefits than the latter one, the Urban and Rural Residents Basic Medical Insurance (URRBMI) has been launched in China since 2009 as an integrated program for merging the different social health insurance programs for both rural residents (i.e., NRCMS) and urban non-working residents (i.e., URBMI) [22]. Although 96% of the Chinese population have been covered by different types of medical insurance programs, large disparities embedded in economic development levels among different administrative regions across the nation has induced non-ignorable inequity for nationwide residents under the coverage of different types of medical insurance programs in terms of accessing health care as well as seeking financial protection via reimbursement procedures to avoid medical expenditure-induced poverty [22, 23]. Another notable factor leading to this inequity would be the variations embedded in health insurance reimbursement rates for inpatient and outpatient services with the same medical insurance program. Taking the reimbursement ratios provided by the NRCMS for its beneficiaries as an example, the reimbursement rate for inpatients is much higher than that of outpatients, while outpatient expenditures are only covered in particular counties [24].

However, few studies from previous literature managed to investigate the factors related to health services underutilization among chronic disease patients, especially for economically disadvantaged population groups living in poverty-stricken regions in both low and middle-income countries (LMICs). Poor accessibility to medical services remains a critical issue in impoverished areas where evenly distributed healthcare resource allocations are difficult to be achieved due to underdeveloped economic development status. In addition, compared with people residing in economically developed regions, residents living in impoverished areas typically have different cultural backgrounds and are at lower educational levels, thus leading to other region-specific characteristics that would potentially compromise efficient utilization of health services. As various factors potentially related to health services underutilization in impoverished regions remain poorly understood, while few studies have explored residents’ self-reported reasons for health services underutilization in economically disadvantaged areas, the identification of critical strategies for achieving improved health services utilization in impoverished regions remains a challenging issue to be addressed at health administrative levels throughout policy-making procedures.

In an attempt to bridge these gaps embedded in previous literature, in this study, we investigated the prevalence of health services underutilization as well as the associated factors in China via selecting disadvantaged population groups living in rural central China as the sample for analysis, among which patients diagnosed with hypertension and diabetes were selected for analysis as the most commonly diagnosed chronic diseases in China. Our study’s findings were expected to provide evidence-based implications for assisting policy-makers in implementing key strategies to minimize health services underutilization in impoverished regions, thus further improving nationwide management of chronic diseases and the associated prognoses, ultimately reducing medical-cost-induced poverty for economically disadvantaged population groups.

## Data and methods

### Data acquisition and study population

#### Data collection

In this study, data were obtained from the 2019 cross-sectional survey, known as the baseline survey before implementing the project (the project name has to be anonymous for confidentiality issues) funded by the Bill & Melinda Gates Foundation and supervised by the National Health Commission of China. The questionnaire was adapted from the Sixth China National Health Service Survey. The questionnaire had been finalized through several rounds of expert consultations and discussions, and a preliminary survey was conducted to further improve the comprehensibility of the questionnaire.

All the interviewers were recruited from two universities in China. They had been trained to follow the interview protocol prior to the interviews in order to ensure all interviewees were informed about our study purposes while their privacy was protected throughout the interviews. Interviewers asked questions in a previously designed sequence, and the answers were recorded in a standardized format for securing the data collected on obtaining participants’ consents.

Answers to a list of questions were collected from participants via face-to-face interviews recorded as multiple covariates for further analysis, including demographics, social structure, income, medical insurance coverage, regular source of care, distance to the nearest health facility, and self-rated score on health conditions. The variable of interest was whether or not the participants had experienced health services underutilization. Records of the questionnaire were cross-checked by two independent interviewers post the survey.

#### Study population

All participants were aged 18 years old and above. A multi-stage cluster sampling method was applied to select the samples. Specifically, three provinces located in central China were selected initially by the National Health Commission of China considering their representativeness of the nationwide situation in terms of the population size, economic development level and geographical location. Within each province, this process was followed by the selection of a particular county based on its capacity for actually implementing the project, its representativeness of the socio-economic development level for that particular province it has been located within as well as its geographic location. After the selection of an intervention county in each province selected, another county with comparable economic level was selected from the same province for comparison purposes. As the results, a total of six counties were selected for analysis in this study, including five identified poverty-stricken counties and one non-poverty-stricken county. Within each county, three townships were selected based on their representativeness of varied levels of economic development status across that particular county, geographical locations, and the total number of respondents available. Within each township, a number of villages were selected considering geographical adjacency to facilitate patients’ recruitment. As the last step, interviewees diagnosed with HBP or DM at county-level health centers were recruited by local village doctors as participants in this study from five poverty-stricken counties. Qualified participants were recruited by village doctors to conduct face-to-face interviews in township health centers. A small number of interviewees who were not able to come due to inconvenience were interviewed in their own homes. After data cleaning, 1559 observations with HBP and 955 observations with DM without missing values on critical variables were retained for analysis.

### Theoretical framework and variables measurement

#### Dependent variables

According to the four-stage continuum of care summarized by Glasziou, et al. [1], we assessed the health services underutilization which occurred during the first stage. At this stage, patients may not be able to access potentially beneficial healthcare, due to the inadequacy of the health care provision, or the inability of patients to reach or to afford medical services available, or both [1]. In this study, health services underutilization was assessed by the question, “Have you ever needed to see a doctor for HBP (for hypertensive patients) or DM (for diabetic patients) but did not go there in the past month?” If the respondent’s answer was yes, it indicated that there were health services underutilization; otherwise, there was no health service underutilization. One was assigned as the value for the variable indicating health services underutilization, and zero was assigned as the value indicating the opposite situation.

#### Independent variables

We applied Andersen’s behavior model to account for independent variables. Multiple studies from previous literature have examined factors affecting health service utilization as the most behavior-affected and demand-side-centered problem [25], among which Andersen’s behavior model [26] has been widely adopted for analysis [27]. The original version of the model was developed in the 1960s [27, 28]. As a conceptual framework, this model suggested that three fundamental dynamics would determine healthcare utilization, namely predisposing factors (predisposition to use services), enabling factors (enabling or impeding use), and need factors (perceived and evaluated need to care) at both individual and contextual levels [26, 27, 29]. At the personal level, (1) the predisposing factors include demographic (biological imperatives, e.g., age, gender, ethnicity) and social structure factors (e.g., marital status, education level, occupation) as well as health beliefs (e.g., attitudes, values, and knowledge related to health and health services which might influence their perceptions for the need and utilization of medical services) [26, 27]; (2) the enabling factors component encompasses both individual-specific resources (e.g., income level, insurance coverage, regular source of care) as well as the healthcare resource allocations among the community they reside in (e.g., the number of health professionals and hospital bed supply) [27]; (3) the need factors are differentiated between perceived need (i.e., how people view and experience their health and disease symptoms) and evaluated need (i.e., professional assessments and objective measurements of patients’ health status and self-reported need for care) [30]. At the contextual level, (1) predisposing factors include the demographic and social compositions of communities, collective and organizational values, cultural norms, and political perspectives [30]; (2) the enabling factors encompass the resources available within the community, the amount, varieties, locations, structures and distribution of health services facilities and personnel, as well as health policies [30]; (3) the need factors are discriminated between environmental need characteristics (i.e., factors reflecting the health-related conditions embedded in the environment) and population health indices (overall community health measures, i.e., epidemiological indicators of mortality, morbidity, and disability) [30].

This model can be applied to assess the equity of health services utilization, which assumes that in the healthcare system where residents’ accessibilities to medical services are equal, it is need factors rather than predisposing or enabling factors that primarily contribute to the variations in health services utilization among different subgroups [27]. This model also distinguishes relatively modifiable components (e.g., health beliefs and enabling factors) from unmodifiable attributes (e.g., demographic and social structural characteristics) [26]. Later versions of this model developed in the 1970s were updated via incorporating the significant impacts of organizational and financial factors on the distribution and delivery of medical services [26, 27]. The third phase of this model, more recently modified (during the 1980s and 1990s), highlighted the impacts of other factors that might result in healthcare disparities while evaluating the effects of health services utilization. After modifications, it is now understood and accepted that individuals’ health behaviors, such as self-care, would primarily affect health services utilization [27].

Based on Andersen’s behavioral model, independent variables included indicators reflective of three dimensions, namely predisposing factors, enabling factors, and need elements [26, 27, 30].

1. Predisposing factors

At the individual level, predisposing factors can be demographic characteristics (e.g., age, gender, ethnicity), social structure (e.g., marital status, education level). Demography and social structure information was measured by multiple-choice questions and directly collected from answers. Options for these variables were list in Table 1.

(2) Enabling factors

**Table 1:** List of variables for empirical analysis

Variable	Characteristics
**Dependent variable**	
Health service underutilization	No (Reference); Yes
**Independent variable**	
*Predisposing factors*	
Age	Years (Continuous)
Gender	Female (Reference); Male
Ethnicity	Han ethnic (Reference); Minorities
Marital status	Other (Unmarried/divorced/widowed, reference); Married
Education level	Primary school or below (Reference); Junior high school; Senior high school or above
*Enabling factors*	
Income	Low (Reference); Middle; High
Medical insurance	Uninsured (Reference); URRBMI (including URBMI or NRCMS); UEBMI Others (e.g., commercial insurance)
Regular follow-up (source of care)	No (Reference); Yes
Distance to the nearest health facility	Less than one kilometer (Reference); 1-5 km; More than 5 km
*Need factors*	
Self-reported health score	Points (Continuous)

Enabling factors refer to individual and community resources that might facilitate individuals to obtain health services (e.g., income, health insurance coverage, regular source of care) or impede health services utilization (e.g., distance to the nearest health service center). Income was rated by self-reported data as compared with the respondents’ neighborhood situation. Regular source of care was assessed by asking “Have you received any follow-up service for your chronic disease from the doctor in the last three months?”. The follow-up service should be targeted for hypertension or diabetes. Distance to the nearest health service center was evaluated by asking “How far is the nearest place to visit a doctor from your home?”. All the variables included in the enabling factors were collected via multiple-choice questions, with options available in Table 1.

(3) Need factors

Need factors can be either need perceived by individuals or evaluated by health professionals. In this study, need factors were assessed through subjective health assessment. EQ-5D visual analogue scale was used to assess subjective health status. This scale was derived from the Chinese version of the international standardized questionnaire EQ-5D designed to measure health-related quality of life [31]. The subjects assessed their health status within a range from 0 (the worst health status imaginable) to 100 scores (the best health status possibly reached) [31].

The descriptions of all variables included in the empirical analysis were listed in Table 1.

#### Self-perceived reasons for health services underutilization

If respondents reported not visiting a doctor when feeling necessary, then a single choice question would be further asked to investigate the underlying reason: “Which of the following is the primary reason for your not visiting a doctor?” The options included “afraid of troubles,” “shortage of money,” “lack of time,” “inconvenient transportation,” “holding the belief that the disease would be incurable even after receiving assistance from doctors,” and “others.”

### Statistical analyses

According to the outcome variable, whether or not the respondent had experienced health service underutilization, we divided the overall subjects into two subgroups and conducted the descriptive analyses. Descriptive analyses showed the general information and comparisons between subgroups regarding predisposing factors, enabling factors, and need factors. We presented mean and standard deviations (SDs) for continuous variables and frequencies with percentages for categorical variables. Subgroup comparisons were conducted through the t-test for continuous variables, the Chi-square test, and the Wilcoxon rank-sum test for unordered and ordered categorical variables. Multilevel logistic models were adopted to examine factors associated with health services utilization when controlling for individuals’ nesting within villages [32]. First, we run an empty model to check the intraclass correlation coefficient (ICC) for multilevel strategy appropriateness. Next, we fitted a random intercept model to allow the log-odds of outcomes to vary from different villages [32].

As described above, these statistical analysis procedures were conducted separately for both patients with hypertension and diabetes. For each type of chronic disease, we also conducted subgroup analyses according to respondents’ poverty status.

Two-sided *p* values less than 0.05 were defined as statistically significant. All statistical analyses were performed in Stata 16 (StataCorp LP, College Station, Texas).

## Results

### Health services underutilization for hypertensive patients

#### Descriptive statistics

Table 2 presents the descriptive analysis results for hypertensive patients. The analytical sample included 1559 respondents, among which 274 (17.58%) reported not visiting a doctor when feeling necessary. The respondents’ average age was 64.83 years (SD: 9.20 years), among which than half (64.53%) were female, and 74.41% were of Han ethnic identity. Nearly four-fifths (79.99%) of respondents were married, and only 9.32% of the respondents received senior high school education or higher levels. In terms of economic status, 34.64% reported low income, and 22.19% reported middle income. Medical insurance programs covered 99.94% of respondents. More than four-fifths (83.42%) had a regular source of care. In terms of accessibilities to medical services, 61.58% could access the nearest health facility within one kilometer, while about 97.50% within five kilometers. The mean self-reported health score was 54.66 points (SD: 22.22 points). Based on whether or not participants had experienced health services underutilization, subgroup comparison outcomes indicated that health services underutilization was more likely to occur among female participants, participants without a regular source of care, and low self-reported health status.

**Table 2:** Descriptive statistics for hypertensive patients

Characteristics	Total (***N*** = 1559)	Health service underutilization		Statistics	***P***-value
		Yes (***N*** = 274)	No (***N*** = 1285)		
***Predisposing factors***					
Age (years)	64.83 (9.20)	64.03 (8.60)	65.01 (9.31)	1.603	0.109
Gender				4.465	0.035
Female	1006 (64.53)	192 (70.07)	814 (63.35)		
Male	553 (35.47)	82 (29.93)	471 (36.65)		
Ethnicity				3.419	0.064
Han	1160 (74.41)	216 (78.83)	944 (73.46)		
Minority	399 (25.59)	58 (21.17)	341 (26.54)		
Marital status				0.130	0.719
Other	312 (20.01)	57 (20.80)	255 (19.84)		
Married	1247 (79.99)	217 (79.20)	1030 (80.16)		
Education attainment				2.259	0.024
Primary school or below	964 (61.95)	186 (67.88)	778 (60.69)		
Junior high school	447 (28.73)	68 (24.82)	379 (29.56)		
Senior high school or above	145 (9.32)	20 (7.30)	125 (9.75)		
***Enabling factors***					
Income level				2.828	0.005
Low	540 (34.64)	121 (44.16)	419 (32.61)		
Middle	346 (22.19)	47 (17.15)	299 (23.27)		
High	673 (43.17)	106 (38.69)	567 (44.12)		
Medical insurance				9.985	0.019
No	1 (0.06)	0 (0.00)	1 (0.08)		
Others	14 (0.90)	6 (2.19)	8 (0.62)		
URRMI	1508 (96.73)	266 (97.08)	1242 (96.65)		
UEBMI	36 (2.31)	2 (0.73)	34 (2.65)		
Regular source of care				11.042	0.001
No	258 (16.58)	64 (23.36)	194 (15.13)		
Yes	1298 (83.42)	210 (76.64)	1088 (84.87)		
Distance to the nearest health facility				−0.553	0.580
Less than one kilometer	960 (61.58)	165 (60.22)	795 (61.87)		
1-5 km	560 (35.92)	101 (36.86)	459 (35.72)		
More than 5 km	39 (2.50)	8 (2.92)	31 (2.41)		
***Need factors***					
Self-reported health score	54.66 (22.22)	45.33 (21.11)	56.64 (21.96)	7.769	< 0.001

#### Multilevel logistic regression results

**Fig. 1:**
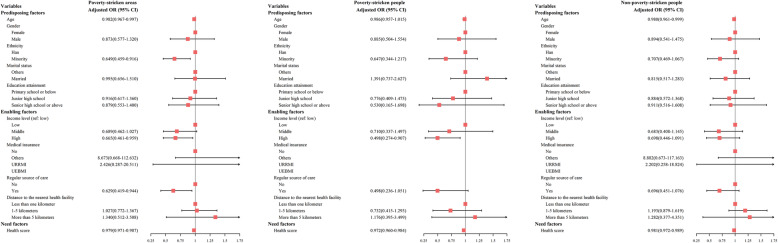
Factors associated with health services underutilization for hypertensive patients

Figure 1 illustrates the multilevel logistic regression results for hypertensive samples. For participants living in poverty-stricken areas, age was found to be significantly associated with health services underutilization, indicating that younger adults tended to underutilize health services more frequently (AOR: 0.982, 95% CI: 0.967-0.997). In addition, ethnic minorities were found to be less like to avoid necessary health services (AOR: 0.649, 95% CI: 0.459-0.916), indicating that residents among the Han ethnic group were 1.5 times more likely to resist against health services when necessary. Respondents who had perceived their income levels as higher than their counterparts demonstrated to be less likely to underutilize health services (AOR: 0.665, 95% CI: 0.461-0.959). Similarly, respondents having a regular source of care had lower odds of health services underutilization than those without having a regular source of care (AOR: 0.629, 95% CI: 0.419-0.944), indicating that respondents not having a regular source of care were approximately 1.6 times more likely to underutilize health services. Participants with better self-perceived health conditions presented to have a lower probability of health service underutilization (AOR: 0.979,95% CI: 0.971-0.987). For impoverished residents in the studied areas, income was found to be significantly associated with the probability of health services underutilization (high-income level: AOR: 0.498, 95% CI: 0.274-0.907). This result suggested that respondents who perceived their income as lower than their counterparts were twice more likely to underutilize health services. Besides, better self-perceived health condition was found to be protective for health services underutilization (AOR: 0.972, 95% CI: 0.960-0.984). For non-impoverished residents, only older age and better self-reported health status were found to be protective factors for health services underutilization.

#### Reasons for health services underutilization

A list of factors was reported as primary reasons for health services underutilization among hypertensive respondents, including other reasons, lack of money, afraid of having troubles, and a shortage of time. More specifically, a quarter of respondents (25.37%) reported other reasons as the primary cause. Shortage of money was declared as the second leading factor for not attending a doctor (23.53%), followed by a lack of time (16.54%) and afraid of having troubles (16.54%).

### Health services underutilization for diabetic patients

#### Descriptive statistics

Table 3 shows the descriptive analysis results for diabetic patients. The analytical sample included 955 respondents, among which 142 (14.87%) reported not visiting a doctor when necessary. The average age of diabetic participants was 63.26 years (SD: 8.84 years). More than half (67.33%) were female, and 73.93% belonged to Han ethnic identity. More than four-fifths (81.68%) were married\, while only 11.01% of the respondents received an education of senior high school or higher levels. 36.75% reported low income compared with their neighborhoods, and 21.57% reported middle income. 99.79% of respondents were covered by medical insurance programs, while more than four-fifths (84.28%) had a regular source of care. In terms of accessibility to medical services, 60.42% could access the nearest health facility within one kilometer and about 95.92% within five kilometers. The mean self-reported health score was 53.16 points (SD: 22.79 points). Subgroup comparison results indicated that health service underutilization was more likely to occur among younger participants, those without a regular care source, or those with poor self-evaluated health status.

**Table 3:** Descriptive statistics for diabetic patients

Characteristics	Total (***N*** = 955)	Health service underutilization		Statistics	***P***-value
		Yes (***N*** = 142)	No (***N*** = 813)		
***Predisposing factors***					
Age (years)	63.26 (8.84)	61.58 (7.73)	63.55 (9.00)	2.453	0.014
Gender					
Female	643 (67.33)	102 (71.83)	541 (66.54)	1.536	0.215
Male	312 (32.67)	40 (28.17)	272 (33.46)		
Ethnicity				1.557	0.212
Han	706 (73.93)	111 (78.17)	595 (73.19)		
Minority	249 (26.07)	31 (21.83)	218 (26.81)		
Marital status				0.000	0.996
Other	175 (18.32)	26 (18.31)	149 (18.33)		
Married	780 (81.68)	116 (81.69)	664 (81.67)		
Education attainment				0.025	0.980
Primary school or below	559 (58.60)	84 (59.15)	475 (58.50)		
Junior high school	290 (30.40)	41 (28.87)	249 (30.67)		
Senior high school or above	105 (11.01)	17 (11.97)	88 (10.84)		
***Enabling factors***					
Income level				2.852	0.004
Low	351 (36.75)	69 (48.59)	282 (34.69)		
Middle	206 (21.57)	25 (17.61)	181 (22.26)		
High	398 (41.68)	48 (33.80)	350 (43.05)		
Medical insurance				10.614	0.014
No	2 (0.21)	1 (0.70)	1 (0.12)		
Others	11 (1.15)	5 (3.52)	6 (0.74)		
URRMI	913 (95.60)	133 (93.66)	780 (95.94)		
UEBMI	29 (3.04)	3 (2.11)	26 (3.20)		
Regular source of care				10.028	0.002
No	150 (15.72)	35 (24.65)	115 (14.16)		
Yes	804 (84.28)	107 (75.35)	697 (85.84)		
Distance to the nearest health facility				−0.009	0.993
Less than one kilometer	577 (60.42)	85 (59.86)	492 (60.52)		
1-5 km	339 (35.50)	53 (37.32)	286 (35.18)		
More than 5 km	39 (4.08)	4 (2.82)	35 (4.31)		
***Need factors***					
Self-reported health score	53.16 (22.79)	46.73 (20.53)	54.29 (22.99)	3.671	< 0.001

#### Multilevel logistic regression results

**Fig. 2:**
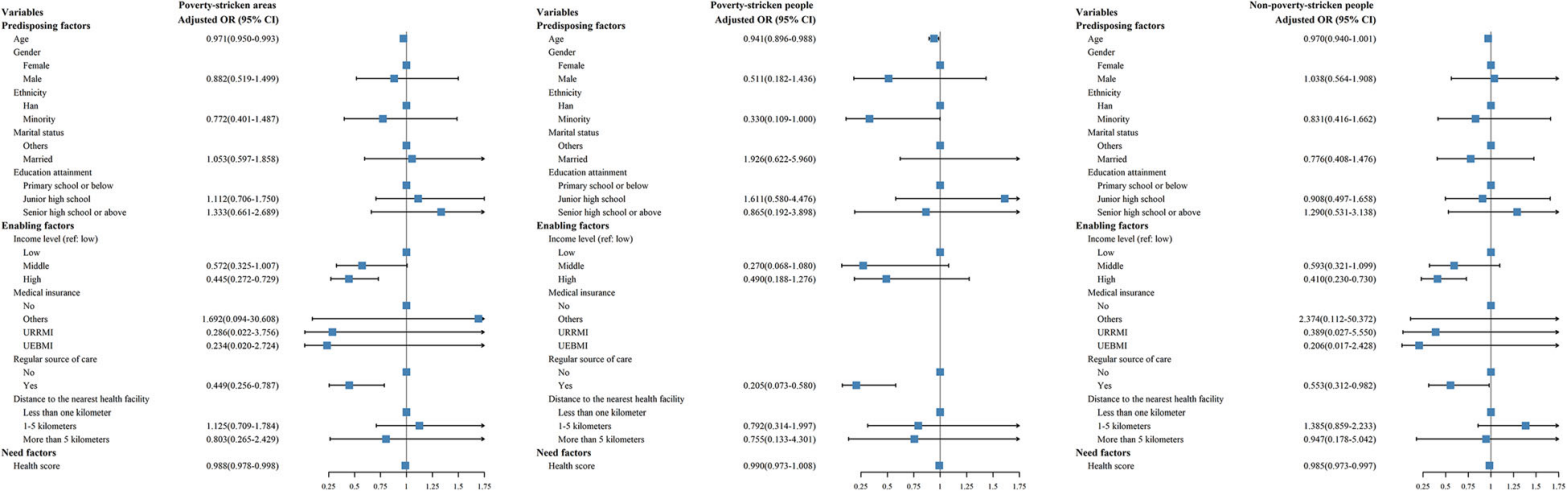
Factors associated with health services underutilization for diabetic patients

Figure 2 presents the multilevel logistic regression results for diabetic participants. For the respondents in poverty-stricken areas, both older age (AOR: 0.971, 95% CI: 0.950-0.993) and higher-income status (AOR: 0.445, 95% CI: 0.272-0.729) were found to be protective factors for health services underutilization. Not having a regular source of care presented as a risk factor for health services underutilization (AOR for having a regular care source: 0.449, 95% CI: 0.256-0.787), which indicated that this group of people were more than twice more likely to underutilize health services. Respondents with higher self-reported health scores presented to be less likely to underutilize health services (AOR: 0.988, 95% CI: 0.978-0.998). For impoverished diabetic patients, we found that older age, having a regular source of care, and better self-perceived health status were protective factors for health services underutilization, which demonstrated to be consistent with findings from overall diabetic patients in impoverished areas. Besides, we found that residents of minority ethnic groups were less likely to underutilize health services (AOR: 0.330, 95% CI: 0.109-1.000), indicating that Han Chinese people were three times more likely to resist against necessary health services utilization than minority ethnic groups. Impoverished diabetic patients without a regular care source were approximately five times more likely to underutilize health services (AOR for those with a regular source of care: 0.205, 95% CI: 0.073-0.580). However, no significant association was identified between income level and health services underutilization. For non-poverty-stricken people, higher income, having a regular source of care, and better self-perceived health were protective factors for health services underutilization.

#### Reasons for health services underutilization

A list of factors was asked among diabetic patients in an attempt to identify the primary reasons for health services underutilization, including lack of time, shortage of money, others, and holding the belief that DM was curable even after seeing a doctor. Based on data collected from respondents, approximately one-third of respondents (29.08%) reported a shortage of time as the leading cause. Lack of money was declared as the second most important reason (23.53%), followed by other reasons not specified in the responses (19.86%) as well as believing that seeing a doctor would be useless (11.35%).

## Discussions

Illness has been highlighted as a significant cause of poverty in the global context. It was estimated that 97 million people, accounting for 1.4% of the world population, were forced into poverty due to health-related out-of-pocket expenditure [33]. In China, 42.2% of all poverty-stricken households were poverty-stricken or pushed back into poverty due to illness. Furthermore, this percentage raised to 44.1% in 2015, indicating that ill-health has become the leading cause of impoverishment in China [34]. Among all the illnesses, chronic diseases have posed an exceptionally weighty financial burden on residents as chronic diseases typically require long-term medical care for management which would broadly restrict patients’ daily activities, thus leading to various problems such as loss of independence, mental sufferings, disabilities or even death [35]. Lan et al. [36] examined the relationship between the possibility of household poverty and the percentage of family members suffering from chronic diseases. They reported that the households demonstrated increased vulnerability to poverty status as this percentage raised due to medical-cost-induced financial burden. Also, rural residents whose family members suffered from chronic diseases were more vulnerable to poverty due to medical-cost-induced financial burden than urban residents [36]. Therefore, chronic diseases have been addressed as leading factors for the economically disadvantaged group worldwide, especially for those living in LMICs [35].

It should be noted that chronic diseases could also be induced by poverty status [37]. Specifically, economically disadvantaged people tend to be more vulnerable to chronic illnesses for multiple reasons, including greater exposure to risks (e.g., smoky fuels [38], tobacco smoking [39], excessive alcohol consumption [40], etc.) [41], poor accessibility to health services [42], inadequate education, poor social networking, social isolation, and long-lasting mental stress [37, 43]. Population living in disadvantaged areas might also face a shortage of qualified healthcare professionals, medicine, and medical equipment, which would become a massive obstacle for them in obtaining medical services when necessary, in a timely manner [37, 42]. Given the mutually-affected relationship between poverty status and chronic diseases, the reduction of chronic disease patients in impoverished regions or at the disadvantaged economic situation has been emphasized as an essential strategy for achieving the reduction of poverty in a worldwide range through preventing chronic disease sufferers from getting trapped into these vicious cycles [37].

Despite those factors associated with health services utilization have continuously been investigated by researchers with impressive findings, this remains a critical issue to be further explored. The reason may lie in that most studies from previous literature have been merely focused on specific types of health services utilization, such as outpatient services [44, 45], thus failing to account for overutilization or unnecessary utilization of health services [19, 46]. As a result, an increased number of researchers began to explore factors associated with health services underutilization as a meaningful approach to identifying the barriers embedded in obtaining access to health services, especially for disadvantaged population groups in need [4, 9, 47, 48].

### Health services underutilization rates in impoverished areas

On average, 17.58% of the respondents diagnosed with HBP had experienced health services underutilization during 1 month before the survey. The rate was 14.87% for diabetic patients. Both rates were found to be lower than estimates from the China Family Panel Studies in 2016 (21.84-29.30%) [19]. One possible reason is that this study [19] estimated this rate for the overall population instead of focusing on people living in impoverished areas [19]. The study also found that the poorer quartiles’ health service underutilization rate was lower than the wealthier quartiles [19]. This result indicated that the rate would be lower in more impoverished areas than more affluent areas because poor neighborhoods are more likely to be economically disadvantaged. Therefore, our results were supported by the findings from Ta et al.’ study [19].

Despite that the rate of health services underutilization for hypertensive patients was found to be higher than for diabetic patients (17.58% vs. 14.87%), no statistically significant differences were found between those rates. We also did not observe any statistically significant difference between poor and non-poor residents diagnosed with two types of studied chronic diseases regarding their health services underutilization rates. This result indicated that health services underutilization for both chronic disease conditions in impoverished regions demonstrated equally prevalent regardless of residents’ economic status.

As previously mentioned in the methodology section, we assessed health services underutilization that occurred in the first stage of the continuum of care. However, the underutilization of health services could also occur at any following stages throughout the continuum of care, which would be reflected as patients’ inaccessibility to potentially effective medical services, the incapacity of doctors for providing both affordable and efficient interventions, or patients’ failure to adhering to interventions as prescribed by doctors [1]. This therefore has highlighted the important role of improving the quality or perceived quality of health care delivered at the primary healthcare level in the process of addressing underutilization issues related to health services provision [1]. Meanwhile, under-diagnosis or under-management associated with unsatisfied quality of care was also unignorable. For this study, it should be noted that the prevalence of health services underutilization might have been under-estimated due to the accumulation of multiple problems from each stage throughout the health-care continuum [1].

### Factors associated with health services underutilization in poor areas

We found that predisposing factors (age), enabling factors (income and regular source of care), and need factors (self-reported health score) were the common predictors of health service underutilization both for hypertensive and diabetic patients in impoverished areas. These factors indicated the existence of inequity embedded in health services utilization.

#### Predisposing factors

The probability of underutilizing health services was found to be relatively low among aged adults, which was consistent with findings from Bovet et al.’, which analyzed health services underutilization in a relatively impoverished area among urban population groups in Tanzania [16]. As reported by this study, 66% of hypertensive patients who were advised to seek health care failed to attend a doctor. On the contrary, younger age was reported as a significant risk factor for health services underutilization [16]. One possible explanation for these findings was that aged population groups tend to avoid delays in seeking medical assistance due to worsening health conditions induced by the aging process, increasing their anxiety levels towards self-perceived health conditions. It is noteworthy that the impact of aging on health services utilization remains ambiguous based on previous literature in this field, which might have been induced by different target groups and various types of health services engaged for analysis [29, 45, 49, 50]. As such, the association between aging and health services utilization needs to be further explored by future studies to provide practical implications for policy-makers, especially for those in countries with significantly aging population structures such as China [29, 51].

Educational level has been widely adopted as an indicator reflective of was participants’ health literacy, which has been expected to be positively associated with the appropriateness of decisions made on health services utilization. However, this study found that participants’ educational levels were unexpectedly not associated with health services underutilization. One possible reason was that residents’ educational levels were commonly low in impoverished areas despite small disparities embedded in participants’ educational levels [16].

#### Enabling factors

Respondents with higher self-rated income were found to be less likely to experience health services underutilization. One possible explanation for this was that residents’ awareness of seeking healthcare services as a fundamental approach for health maintenance tends to be raised with increased income [27]. On the contrary, the efficiency of health services utilization for residents with inadequate income might be potentially compromised by multiple factors such as lack of money, stress, and schedule conflicts induced by labor. It is noteworthy that patients diagnosed with chronic diseases would be suggested to stick with specific therapy plans prescribed by doctors, such as insulin injections to control blood sugar levels for diabetic patients. However, evidence from previous literature indicated that even in urban diabetic centers, a quarter of patients demonstrated insulin underutilization and unfavorable glycemic control due to sensitivity to medical costs [52]. This result was found to be consistent with our findings that approximately one out of four diabetic respondents reported health services underutilization as the result of sensitivity to medical costs, which highlighted the affordability of diabetic therapy plans as a critical aspect to be considered in achieving chronic disease management from a long-term perspective. Although the penetration rate of different social health insurance schemes in a nationwide range presented to be high, these schemes’ benefits packages merely provided limited or even no reimbursement for outpatient services [24]. This could explain the insignificance of health insurance schemes’ association with underutilization of health services as suggested by our findings as our study mainly focused on assessing outpatient services.

After controlling for all the other covariates, regular sources of care were a relatively decisive protective factor for health services underutilization in impoverished areas among hypertensive and diabetic patients. Such finding has highlighted the positive impact of obtaining regular sources of healthcare on facilitating residents’ appropriate utilization of health services. China has implemented the national basic public health service (BPHS) program since 2009, in which chronic disease (hypertension and type II diabetes mellitus) management has been addressed as an essential part [53]. According to rules and regulations for chronic disease management, primary health workers are required to provide four follow-up visits each year for residents diagnosed with any one type of these chronic diseases [53]. These follow-up visits make up a regular source of care. For patients with HBP, a series of medical services will be provided throughout follow-up visits, including measuring blood pressure, evaluating whether there is a critical situation and providing follow-up referral services when necessary; monitoring body weight, heart rate, and body mass index; asking patients about their disease-affected lifestyles as well as assessing patients’ medication compliance. For patients with DM, the follow-up services typically include measurement of fasting blood glucose and blood pressure, assessment of critical conditions and provision of referral services if necessary, measurement of body mass index (BMI), an examination of dorsal foot pulse, asking about disease-affected lifestyles as well as evaluating patients’ medication compliance.

These services have been provided as part of follow-up visits in an attempt to minimize health services underutilization among patients suffering chronic diseases in impoverished regions via facilitating timely access to medical services at residents’ own perceived needs for seeking healthcare. Specifically, objective evaluations on patients’ health conditions conducted by healthcare professionals during follow-up visits would significantly improve patients’ knowledge about their diseases, thus stimulating self-perceived needs for the medical assessment for chronic disease management in the long term [30]. Also, health education provided by healthcare professionals during follow-up visits would assist patients in coping with their diseases in an appropriate way. Another aspect that patients could potentially benefit from those follow-up visits is that appointments can be made for out-clinic consultations as needed. All these services provided during follow-up visits would likely help economically disadvantaged population groups overcome communication barriers, relieve social isolation, and facilitate individuals’ social engagement, thus ultimately achieving improved utilization of health services necessary for residents [54].

Based on our findings, regular follow-up visits were found to have decisive protective effects on the underutilization of health services for patients diagnosed with chronic diseases. However, approximately 16% of chronic disease patients living in impoverished rural areas have no access to this kind of regular healthcare service, which should be addressed as a critical issue for policy-makers at health administrative levels. In impoverished rural areas in China, village doctors are the primary providers of basic public health services and are responsible for managing chronic disease patients within their service ranges [55]. Previous studies have suggested a series of strategies for improving chronic disease management as an essential part of basic public health services, which specifically included the provision of training programs tailored for the specific needs of village doctors, the adoption of financial incentives, the implementation of high-level integrated management approaches as well as the initiation of the New Rural Cooperative Medical Scheme (NRCMS) contracting methods [55–57]. However, studies are still needed to validate these findings further and identify effective measures for improving chronic disease management in impoverished areas.

#### Need factors

Self-reported health score was found to be negatively associated with health services under-utilization. One possible explanation for this outcome was that respondents with better-self-evaluated health status tend to be more confident in benefitting from health services utilization.

### Self-perceived reasons for health services underutilization

For respondents with HBP or DM, the lack of time and money was reported as the primary reasons for not attending a physician when the respondents felt it necessary. These findings are expected to assist policy-makers in adopting critical strategies for improving the accessibility to healthcare services for patients with chronic diseases. The provision of affordable medical services and medications should be addressed as an important goal [52]. Another goal would be to balance suppliers and demanders in primary care by eliminating the spatial barriers for obtaining healthcare services targeted at chronic disease management [58–60]. However, it should be noted that the proportion of respondents reporting “other reasons” for underuse was substantial while not specified. Future studies exploring this point should be more informative in order to facilitate the adoption of evidence-based strategies in an effective manner.

### Limitations of the study

Several limitations should be noted when interpreting our findings. First, the lack of randomness and the limited sample size should be considered the flaws embedded in the process of sample collection [61]. Second, samples recruited in this study were selected from central China, thus it should be cautious to generalize the findings to other parts of China, such as the western or northeastern regions. Third, our study was based on a cross-sectional survey, for which the causal inferences should be avoided [62], which means that the direction of the causality inherent in the relationship between health services underutilization and self-perceived health status remains to be further validated. Fourth, as retrospective interviews were adopted in our study as the method for identifying exposures, biased outcomes might be induced due to multiple potential factors such as different time intervals during interviews, information details reported by interviewees, as well as a list of items used in the survey associated with participants’ expectations for social well-being [63]. Therefore, it is highly recommended that both intervention and comparative studies be conducted more comprehensively in future studies as an improved approach for validating the causality embedded in the relationship between health services underutilization and multiple underlying factors among patients with chronic diseases.

## Conclusions

In conclusion, this study described health services underutilization for chronic disease patients in poverty-stricken areas and identified its associated factors. To the best of our knowledge, this is the first study to investigate health services underutilization and its related factors for chronic disease patients in poverty-stricken areas in China, which is expected to shed light on future research in this area. Based on the outcomes, a list of predisposing factors (age), enabling factors (income and regular source of care), and need factors (self-reported health score) were found to be the common predictors of health service underutilization both among hypertensive and diabetic patients in studied areas. Therefore, comprehensive strategies should be addressed throughout policy-making procedures to improve nationwide penetration of basic public health services, thus ultimately facilitating the equity of health services utilization in a national range.

## Supplementary Information

**Additional file1.**

## Data Availability

All data generated or analyzed during this study are included in this published article.

## Abbreviations

HBP: High blood pressure, hypertension
DM: Diabetes mellitus
GBD: Global Burden of Diseases
UI: Uncertainty Interval
DALYs: Disability-adjusted Life Years
LMICs: Low and Middle-income Countries
BP: Blood pressure
URRBMI: The Urban and Rural Residents Basic Medical Insurance
URBMI: The Urban Resident Basic Medical Insurance
NRCMS: The New Rural Cooperative Medical Scheme
UEBMI: The Urban Employee Basic Medical Insurance
SD: Standard Deviation
SE: Standard error
OR: Odds ratio
AOR: Adjusted odds ratio
CI: Confidence interval
ICC: Intraclass Correlation Coefficient
