# Supplementary material for: Health service underutilization and its associated factors for chronic diseases patients in poverty-stricken areas in China: a multilevel analysis
Source: BMC Health Serv Res. 2021 Jul 18;21:707. doi: 10.1186/s12913-021-06725-5 (PMC8286576; doi:10.1186/s12913-021-06725-5)
Supplement: Supplementary file 1 — Additional file1. [file 12913_2021_6725_MOESM1_ESM.pdf]

Baseline Questionnaire for China Primary Health Care Impact Evaluation[1]

County code	□□□□□□	Township code	□
Village code	□	Participant code	□□□□

Respondent's information

1. Name:________________________
2. ID number:____________________
3. Telephone number:_____________
4. Address
   County:_______________________
   Town:____________________
   Village:_______________________
5.Chronic disease type:

☐Hypertension ☐Diabetes mellitus ☐Both Hypertension and Diabetes mellitus

[1] The questionnaire was designed for the baseline survey for the research project entitled "China Primary Health Care Impact Evaluation", which was funded by the Bill & Melinda Gates Foundation and led by Professor Winnie Yip. The baseline survey took place during April and May in 2019. The questionnaire was in Chinese and translated into English by the authors of the manuscript.

Table 1. Basic information of the respondent

Question number	Questions and options	Answer
1	What is the relationship of you with the head of the household?<br>1) The same person<br>2) Spouse<br>3) Son or daughter<br>4) Son-in law or daughter-in-law<br>5) Parent<br>6) Parent-in-law<br>7) Grandson or granddaughter<br>8) Brother or sister<br>9) Other	
2	Record of the respondent's gender<br>1) Male<br>2) Female	
3	What is your nationality?<br>1) Han<br>2) Minority	
4	How old are you? (In years)	
5	What is your highest educational attainment?<br>1) Primary school or below<br>2) Junior high school	

Question number	Questions and options	Answer
	3) Senior high school<br>4) Technical secondary school<br>5) College degree or above	
6	What is your marital status?<br>1) Single<br>2) Married<br>3) Widowed<br>4) Divorced	
7	What is your occupation?<br>1) Farmer<br>2) Unemployed<br>3) Part-time job<br>4) Self-employed<br>5) Professionals & technical<br>6) Grassroots staff<br>7) Retired<br>8) Others	
8	Does the following medical insurance cover you?<br>*Notes: Common options for the following are:*<br>*1) Yes*<br>*2) No*<br>I. Urban Employee Basic Medical Insurance (UEBMI)<br>II. Urban Residents' Basic Medical Insurance (URBMI)<br>III. New Rural Co-operative Medical System (NRCMS)	

Question number	Questions and options	Answer
	IV. Urban and Rural Residents Medical Insurance (URRMI)<br>V. Commercial Medical Insurance (CMI)<br>VI. Others	
9	Are you a government medical aid beneficiary?<br>1) Yes<br>2) No	
10	Please rate your overall health today.<br>0 10 20 30 40 50 60 70 80 90 100<br>*Notes: A scale was shown to the respondent. This scale is numbered from 0 to 100. 100 means the best health you can imagine. 0 means the worst health you can imagine.*	
11	Are you smoking?<br>1) Everyday<br>2) Seldom<br>3) Already quit<br>4) Never	
12	Have you drunk any alcohol in the last month?<br>1) At least three times a week<br>2) Less than three times a week<br>3) Never	
About the chronic disease		
Please ask questions numbered 13-26 if respondents had hypertension.		
13	How frequently you take the antihypertensive drug?	

Question number	Questions and options	Answer				
	1) Daily, according to the doctor's request<br>2) Not always, unless necessary<br>3) Never					
14	Have you taken your blood pressure yesterday?<br>1) No<br>2) Yes<br>*Notes: if the respondent answered "Yes," record the blood pressure and skip to question 16*					
15	When was the last time you took your blood pressure?<br>1) Within one week<br>2) Within one month<br>3) Within three months<br>4) Within half a year<br>5) Half a year ago					
16	What has your doctor done about your high blood pressure in the last three months?<br>1) Follow-up<br>2) No follow-up					
17	How many times have you seen a doctor because of high blood pressure in the last month?<br>*Notes: If the record was zero, then skip to question 25.*					
Ask the respondent about the last five outpatient services during the previous month.		1	2	3	4	5
18	What was the first contact health sector?<br>1) Village clinic					

Question number	Questions and options	Answer				
	2) Private clinic<br>3) Town health center<br>4) Public hospital within the county<br>5) Private hospital within the county<br>6) County hospital outside the county<br>7) City or provincial hospital outside the county<br>8) Other					
19	How did you access the health sector?<br>1) On foot<br>2) By bicycle<br>3) By motorcycle<br>4) By car<br>5) Other					
20	How long did you spend on accessing the health sector? (In minutes)					
21	If the doctor said it was uncurable, where did you go to see the doctor later? (Patients receiving treatment in first-visit medical institutions do not need to answer this question)<br>1) Town health center<br>2) Public hospital within the county<br>3) Private hospital within the county<br>4) County hospital outside the county<br>5) City or provincial hospital outside the county<br>6) Other					
22	How much did it cost, including examination, diagnosis, and treatment,					

Question number	Questions and options	Answer				
	medicine, etc. (that is, the total outpatient fee)? (In RMB Yuan)					
23	How much was the out-of-pocket expenditure? (In RMB Yuan)					
24	How much did you and the person who accompanied you spend on this visit (such as bus, food, and accommodation)? (In RMB Yuan)					
25	Have you ever needed to see a doctor because of high blood pressure but didn't see a doctor in the last month?<br>1) Yes<br>2) No<br>*Notes: If the respondent answered "No" and the respondent had diabetes mellitus, skip to question 27; If the respondent answered "No" and did not have diabetes mellitus, skip to Table 2.*					
26	The main reasons for not going to see a doctor (single selection): Seeing a doctor is a troublesome thing<br>1) Shortage of money<br>2) Shortage of time<br>3) Inconvenience concerning approaching the doctor<br>4) The disease cannot be cured<br>5) Others					
Ask questions 27-41 if the respondent had diabetes mellitus.						
27	What is your type of diabetes mellitus?<br>1) Type I<br>2) Type II<br>3) Unknown					
28	How frequently you take antidiabetic drugs (Or take insulin injection)?					

Question number	Questions and options	Answer				
	1) Every day or every meal, according to the doctor's request<br>2) Not always, unless necessary<br>3) Never					
29	Have you taken your blood glucose yesterday?<br>1) No<br>2) Yes<br>*Notes: if the respondent answered "Yes," record the fasting or non-fasting blood glucose, and skip to question 31*					
30	When was the last time you took your blood glucose?<br>1) Within one week<br>2) Within one month<br>3) Within three months<br>4) Within half a year<br>5) Half a year ago					
31	What has your doctor done about your diabetes mellitus in the last three months?<br>1) Follow-up<br>2) No follow-up					
32	How many times have you seen a doctor because of diabetes mellitus in the last month?<br>*Notes: If the record was zero, then skip to Table 2.*					
Ask the respondent about the last five outpatient services during the previous month.		1	2	3	4	5
33	What was the first contact health sector?<br>1) Village clinic					

Question number	Questions and options	Answer				
	2) Private clinic<br>3) Town health center<br>4) Public hospital within the county<br>5) Private hospital within the county<br>6) County hospital outside the county<br>7) City or provincial hospital outside the county<br>8) Others					
34	How did you access the health sector?<br>1) On foot<br>2) By bicycle<br>3) By motorcycle<br>4) By car<br>5) Others					
35	How long did you spend on accessing the health sector? (In minutes)					
36	If the doctor said it was uncurable, where did you go to see the doctor later?<br>1) Town health center<br>2) Public hospital within the county<br>3) Private hospital within the county<br>4) County hospital outside the county<br>5) City or provincial hospital outside the county<br>6) Other<br>*Notes: Patients receiving treatment in first-visit medical institutions do not need to answer this question.*					

Question number	Questions and options	Answer				
37	How much did it cost, including examination, diagnosis, and treatment, medicine, etc. (that is, the total outpatient fee)? (In RMB Yuan)					
38	How much was the out-of-pocket expenditure? (In RMB Yuan)					
39	How much did you and the person who accompanied you spend on this visit (such as bus, food and accommodation)? (In RMB Yuan)					
40	Have you ever needed to see a doctor because of diabetes mellitus but didn't see a doctor in the last month?<br>1) Yes<br>2) No<br>*Notes: If the respondent answered "No", skip to Table 2.*					
41	The main reasons for not going to see a doctor (single selection): Seeing a doctor is a troublesome thing<br>1) Shortage of money<br>2) Shortage of time<br>3) Inconvenience concerning approaching the doctor<br>4) The disease cannot be cured<br>5) Others					

Table 2 Hospitalization utilization

Question number	Questions and options		Answer				
42	Since April 1, 2018, have you been hospitalized by a doctor, but you have not been hospitalized?<br>1) Yes<br>2) No<br>*Notes: If the respondent had been hospitalized during the past 12 months, skip to question 44; otherwise, skip to Table 3.*						
43	What was the main reason for the last time you need to be hospitalized but not hospitalized (single selection)?<br>1) Getting hospitalized is a troublesome thing<br>2) Shortage of money<br>3) Shortage of time<br>4) Inconvenient traffic<br>5) The disease cannot be cured<br>6) Unnecessary for a minor ailment<br>7) No beds available in the hospital<br>8) Indifferent<br>9) Other						
If the respondent has been hospitalized from April 1, 2018, to the present, ask about each hospitalization starting from the most recent hospitalization.		1	2	3	4	5	6
44	Hospitalization year						
45	Hospitalization month						

46	Where were you hospitalized?<br>1) Town health center<br>2) Public hospital within the county<br>3) Private hospital within the county<br>4) County hospital outside the county<br>5) City or provincial hospital outside the county<br>6) Other						
47	How did you access the hospital?<br>1) On foot<br>2) By bicycle<br>3) By motorcycle<br>4) By car<br>5) Others						
48	How long did you spend on accessing the health sector? (In minutes)						
49	Why you discharged from that hospital?<br>1) Already cured, discharge for medical advice<br>2) No cured, transferred for medical advice<br>3) Asking for discharge<br>4) Other<br>*Notes: If the answer was option 1, 2, or 4, skip to question 51.*						
50	Why did you ask for discharge?<br>1) The disease has not been cured<br>2) I feel good enough<br>3) Hospitalization costs too much						

	4) Underequipped hospital<br>5) Poor professional skill<br>6) Other						
51	How much the total charge of hospitalization? (In RMB Yuan)						
52	How much was the out-of-pocket expenditure? (In RMB Yuan)<br>*Notes: Excluding reimbursement and personal medical account expenses*						
53	How much did you and the person who accompanied you spend on this hospitalization (such as taking a bus, accommodation, and hiring a nursing worker)? (In RMB Yuan)						
54	How many days did the family member who accompanied you be absent from work while in the hospital?						

Table 3 Household information

Question number	Questions and options	Answer
55	How many people do you usually eat and live with within your family? (That is, the resident population)	
56	How far is the nearest place to see a doctor from your home?<br>1) Less than one kilometer<br>2) One to five kilometers<br>3) More than five kilometers	
57	How do you handle your minor ailment?<br>1) To see a doctor<br>2) To go to the pharmacy to get over-the-counter (OTC) medicine<br>3) Indifferent<br>4) Other<br>*Notes: If the respondent answered option 2, 3, or 4, skip to question 59.*	
58	Where do you access to doctor's help for a minor ailment?<br>1) Village clinic<br>2) Private clinic<br>3) Town health center<br>4) Public hospital within the county<br>5) Private hospital within the county<br>6) City or provincial hospital outside the county<br>7) Other	
59	Has your family been registered as a poor household?	

	1) Yes<br>2) No<br>3) Unknown<br>*Notes: If the answer was option 2, skip to 61.*	
60	Has your family been alleviated out of poverty?<br>1) Yes<br>2) No<br>3) Unknown	
61	What is your family income in 2018?<br>a. What do you think of your family's income in 2018 compared with the people around you?<br>1) very low 2) low 3) medium 4) high 5) very high<br>b. How much money did your family earn in 2018? (In thousands RMB Yuan)<br>*Notes: Choose one of the two questions above. Total household income includes wage income, net operating income, property income, and transfer income.*	
62	How much did your family spend on daily life in 2018? (That is, household consumption expenditure)<br>Or, ask the percentage of the family spend on daily life relative to total income.	
63	How much did your family spend on food? (Or ask for percentage)	
64	How much did your family spend on medicines, medical services, and products? (Or ask for percentage)	
65	Was your family a subsistence allowance beneficiary?<br>1) Yes	

	2) No<br>3) Unknown	
66	What the main reason for your family being in poverty?<br>1) Lack of labor<br>2) Natural disaster<br>3) Influenced labor cause by disease<br>4) Out-of-pocket expenses related to disease<br>5) Unemployed<br>6) Anthropogenic factor<br>7) Other<br>*Notes: This question is only available for those registered as poor households.*	
67	Are you satisfied with the poverty alleviation?<br>1) Very dissatisfied<br>2) Dissatisfied<br>3) Generally satisfied<br>4) Quite satisfied<br>5) Very satisfied<br>6) Unknown<br>*Notes: This question is available for those registered as poor households.*	

Table 4 Health literacy

Question number	Questions and options	Answer
If the respondent had hypertension and diabetes mellitus, ask questions 68-87; if the respondent had hypertension but without diabetes mellitus, ask questions 68-72 and 80-87; otherwise, ask questions 73-87.<br>*Notes: The interviewer read the sentence and ask about the interviewee's judgment on the sentence. Common options for the following sentences are:*<br>*1) Right*<br>*2) Wrong*<br>3) *Unknown*		
68	High blood pressure is acceptable as long as we don't feel sick.	
69	Not less than 140/90mmHg is considered high blood pressure.	
70	Without treatment, high blood pressure can damage the kidneys.	
71	We can sense our blood pressure.	
72	Hardening or narrowing of blood vessels caused by fatty deposits can cause high blood pressure.	
73	Diabetes is due to insufficient insulin secretion or deficiency.	
74	Diabetes can be cured by taking medicine over some time.	
75	Diabetic patients can eat freely if their fasting blood glucose is normal in the morning.	
76	Prolonged hyperglycemia can cause other health problems, such as blindness.	
77	Normal fasting blood glucose levels do not exceed seven mmol/L.	
78	Diabetic patients can eat fruits ad libitum.	
79	Diabetic patients can share their medicine.	
80	When blood glucose or pressure is controlled, you can stop taking medicine.	

81	All medicines should be taken only after meals.	
82	A double-dosage would compromise a missed medication.	
83	Taking hypoglycemic agents or antihypertensive drugs every two days would reduce adverse reactions.	
84	All the medicine should be kept in the refrigerator.	
85	Smoking increases the risk of heart disease.	
86	Not eating sugar will lead to a lack of energy.	
87	Vegetable oils are cholesterol-free and can be eaten in large quantities.	
